# Anti-Oxidant and Anti-Inflammatory Activities of *Inonotus obliquus* and Germinated Brown Rice Extracts

**DOI:** 10.3390/molecules18089293

**Published:** 2013-08-02

**Authors:** Trishna Debnath, Sa Ra Park, Da Hye Kim, Jeong Eun Jo, Beong Ou Lim

**Affiliations:** Department of Life Science, College of Biomedical and Health Science, Research Institute of Inflammatory Disease, Konkuk University, Chungju 380-701, Korea; E-Mails: trishna_rahul@yahoo.com (T.D); skysarav@naver.com (S.R.P.); junfeel@kku.ac.kr (D.H.K.); whwjddms918@kku.ac.kr (J.E.J.)

**Keywords:** dietary supplement, ROS, antioxidant enzyme, pro-inflammatory cytokine

## Abstract

*Inonotus obliquus* (IO) is parasitic mushroom that grows on birch and other trees in Russia, Korea, Europe and United States. However, IO is not readily available for consumption due to its high cost and difficult growth. In this regard, IO was inoculated on germinated brown rice (GBR) in the present study and the antioxidant and anti-inflammatory activities of the IO grown on germinated brown rice (IOGBR) extracts were evaluated extensively and compared with those for IO and GBR. IOGBR showed highest antioxidant activities with scavenging total intracellular ROS and MDA levels as well as increasing the antioxidant enzymes activity in the H_2_O_2_-stimulated mice liver. It also exhibited best inflammatory activities by suppressing the proinflammatory mediators such as NO, PGE_2_, iNOS, COX-2, TNF-α, IL-1β, and IL-6 in an LPS-stimulated RAW 264.7 cell line. This study provides a comparative approach to find out an excellent natural source of antioxidants and anti-inflammatory agent as a dietary supplement.

## 1. Introduction

Oxidative stress is associated with a variety of chronic inflammatory diseases including arthritis, atherosclerosis, cancer, diabetes, hepatitis, neurodegeneration, and early aging. Oxidative damage is prevented by a defensive system that includes nonezymatic and enzymatic antioxidants [1]. Superoxide dismutase (SOD), catalase (CAT), and glutathione peroxidase (GPx) enzymes are widely used as markers of oxidative stress [2].

Inflammation is a normal physiological immune response that occurs in response to trauma, infection, tissue injury, or noxious stimuli [3,4,5]. In this system, activated inflammatory cells (neutrophils, eosinophils, mononuclear phagocytes and macrophages) increase the secretion of nitric oxide (NO), prostaglandin E_2_ (PGE_2_), and cytokines. Macrophages have three main functions in inflammation—antigen presentation, phagocytosis, and immunomodulation through the production of various cytokines and growth factors. Therefore, macrophages behave as critical players in the initiation, maintenance, and resolution of inflammation. Lipopolysaccharide (LPS) is a major component of the outer membrane of gram-negative bacteria, which has often been used in inflammatory response. In response to LPS, the macrophage secretes various proinflammatory mediators including tumor necrosis factor-α (TNF-α), interleukin-1β (IL-1β), IL-6, NO, and PGE_2_. Overproduction of this mediator is responsible for inflammation. Inhibition of a proinflammatory mediator, therefore, is beneficial in attenuating an inflammatory disorder. 

Mushrooms have been consumed extensively in humans’ daily diet as an supplementary food item since ancient times. They are an excellent source of secondary metabolites, vitamins, minerals, protein, and carbohydrates, as well as high in fiber and low in fat [6]. They also contain various bioactive molecules including terpenoids, steroids, phenols, nucleotides, glycoprotein derivatives, and polysaccharides [7]. Therefore, they have been considered as potential source of antioxidant and anti-inflammatory activity.

*Inonotus obliquus* (IO), commonly known as the chaga mushroom, belongs to the Inonotus genus and the Hymenochaetaceae family of Basidiomycetes. IO is found in the birch forests of Russia, Korea, Eastern and Northern Europe, northern areas and North Carolina Mountains of the USA, and Canada. IO is variously known as “Gift from God” and “Mushroom of Immortality” in Siberia, “Diamond of the Forest,” in Japan and “King of Plants” in China. IO has been considered a traditional medicine in Russia and Eastern European since the sixteenth century [8]. There have been a number of studies exploring its antioxidant, anti-tumor, and antimicrobial activities [9,10,11]. However, IO is not readily available in food diets due to the difficulties of growing it and its high cost. Therefore, in the present study IO was grown on germinated brown rice to increase its availability [12]. Then, IO was extracted with ethanol. In addition, germinated brown rice (GBR) has been soaked in water for 24 h to have germ of approximately 1 mm long [13]. During germination, The brown rice has been enriched extensively with nutrients including γ-aminobutyric acid (GABA), dietary fiber, inositols, ferulic acid, phytic acid, tocotrienols, magnesium, potassium, zinc, γ-oryzanol, and prolylendopeptidase inhibitor [14]. The mushroom grown on germinated brown rice have been found to have an antioxidative role by suppressing the oxidative liver damage and stopping the overproduction of immunoglobulin (Ig) [12,15,16]. In our previous study, we reported the antioxidant activity of extracts of different polarities from IO grown on germinated brown rice (IOGBR); the ethanol extracts showed the highest activity among the three extracts [17]. It is very important to compare the bioactivity of IOGBR with that for GBR, IO. To the best of our knowledge, it is the first comparative study on bioactivity of IO, when it has been grown on germinated brown rice. Therefore, interest has been arisen in investigating and comparing the antioxidant and anti-inflammatory activity of ethanolic extracts of IO, GBR, and IOGBR.

## 2. Results and Discussion

**Figure 1:**
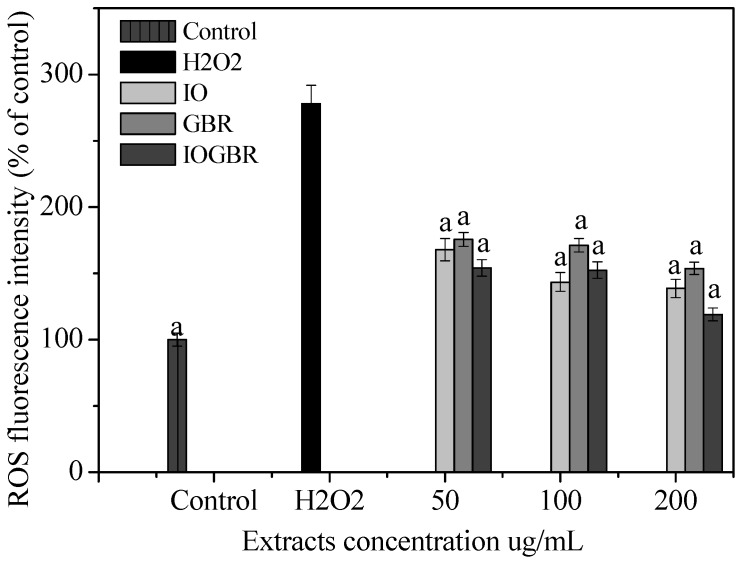
Effect of the extracts from *Inonotus obliquus* (IO), germinated brown rice (GBR) and *Inonotus obliquus* grown on germinated brown rice (IOGBR) on ROS production in H_2_O_2_-treated Chang liver cells.

In the present study, the intracellular ROS was formed within H_2_O_2_-treated liver cells and then treated with the extracts. Figure 1 shows the increasing mean fluorescence intensity in the H_2_O_2_-treated cells along with the control. In contrast, pretreatment with the extract for 1 h significantly decreased the mean fluorescence intensity in a dose-dependent manner. Here, IOGBR showed the highest intensity among the three extracts. Therefore, the highest intensity of IOGBR indicates that it can be an excellent candidate to prevent or reduce diseases related to ROS.

A lipid peroxidation chain reaction can be produced by overproduction of ROS that are responsible for pathological disorders [18]. In peroxidation, ROS mostly affect the polyunsaturated fatty acids (PUFA) in membrane lipids leading to the formation of lipid peroxides such as malondialdehyde (MDA). In the present study, we observed significantly increased levels of MDA in sample B (damage) compared to sample A (control) *p <* 0.05, Table 1). After the extracts treatment, the levels of MDA were significantly decreased in all treated samples (C to F) in a dose-dependent manner compared to the B sample (*p <* 0.05), indicating that all extracts are able to donate electrons and to terminate the lipid peroxidation chain reaction. Furthermore, the capacity of the IOGBR extract was nearly equal to that of the positive control AA (*p >* 0.05; Table 1). The mushroom extracts have capacity to control lipid peroxidation along with improving the antioxidant status, reported by Mishra and Singh [19]. Our results are consistent with the previous report.

**Table 1:** Superoxide dismutase (SOD), glutathione peroxidase (GPx), and catalase (CAT) activities and malondialdehyde (MDA) level with and without the treatment of *Inonotus obliquus* (IO), germinated brown rice (GBR) and *Inonotus obliquus* grown on germinated brown rice (IOGBR) in mice liver damaged by H_2_O_2_. Ascorbic acid (AA) and bovine liver catalase were used as positive control.

Sample	SOD-like activity ^a^ (%)	Catalase activity ^a^ (nmol/min/mL)	GPx activity ^a^ units/mg protein	MDA level ^a^ (mm/min/mg protein)
A (control)	71.95 ± 1.4	5.02 ± 0.2	26.88 ± 2.0	0.547 ± 0.10
B (damage)	37.14 ± 0.5	2.59 ± 0.4	18.80 ± 1.5	2.126 ± 0.06
C (GBR + H_2_O_2_)	41.66 ± 1.5	3.55 ± 0.5	20.18 ± 1.2	1.37 ± 0.05
D (IO + H_2_O_2_)	54.02 ± 2.7	3.90 ± 1.0	22.99 ± 2.0	0.89 ± 0.07
E (IOGBR + H_2_O_2_)	60.51 ± 2.1	4.73 ± 1.5	25.41 ± 2.1	0.712 ± 0.10
F (AA + H_2_O_2_)	68.58 ± 1.8	-	26.41 ± 1.0	0.692 ± 0.15
Bovine liver Catalase	-	4.96 ± 1.2	-	-

^a^ Each value is expressed as mean ± standard deviation (*n* = 3).

Superoxide dismutase (SOD), glutathione peroxidase (GPx), and catalase (CAT) are important to regulate the internal environment and tend to maintain a stable and normal cell function. SOD converts the highly reactive superoxide radical to H_2_O_2_. CAT is present in all body organs, being especially abundant in the liver. It is found in peroxisomes of liver and acts to metabolize hydrogen peroxide to water and diatomic oxygen, while GPx catalyzes the reduction of a variety of hydroperoxides (ROOH and H_2_O_2_) [20,21,22]. In the present study, sample B (damage) decreased the activities of SOD, CAT, and GPx more compared to that of sample A and an increased level of SOD, CAT, and GPx activities were found in all treatment samples (C to F) (Table 1). These results suggested that H_2_O_2_ caused oxidative damage in sample B, which is consistent with the report by Wang *et al.* [23]. They reported that H_2_O_2_ (0.5 mM) induced oxidative stress in a normal human liver cell increased the level of MDA and decreased the antioxidant enzymes. Our findings are in accordance with their study. In addition, IOGBR showed the best activities among the three samples. These properties of IOGBR represent the first line of defense against ROS. The ROS-scavenging power and/or inhibitory activities of natural compounds on lipid peroxidation may, thus, be expected to have therapeutic potential for inflammatory diseases.

**Figure 2:**
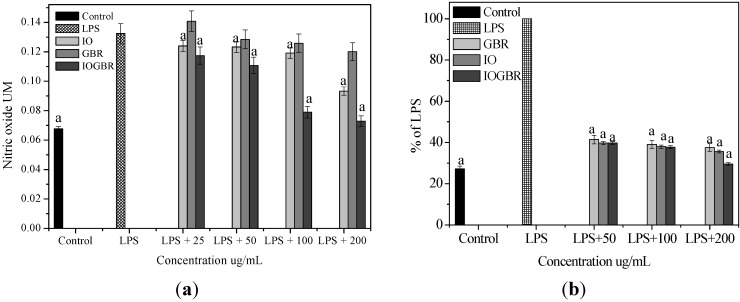
(**a**) Effect of the extracts of *Inonotus obliquus* (IO), germinated brown rice (GBR) and *Inonotus obliquus* grown on germinated brown rice (IOGBR) on LPS-induced NO production Raw 264.7 (**b**) Dose dependent PGE_2_ level of *Inonotus obliquus* (IO), germinated brown rice (GBR) and *Inonotus obliquus* grown on germinated brown rice (IOGBR).

**Figure 3:**
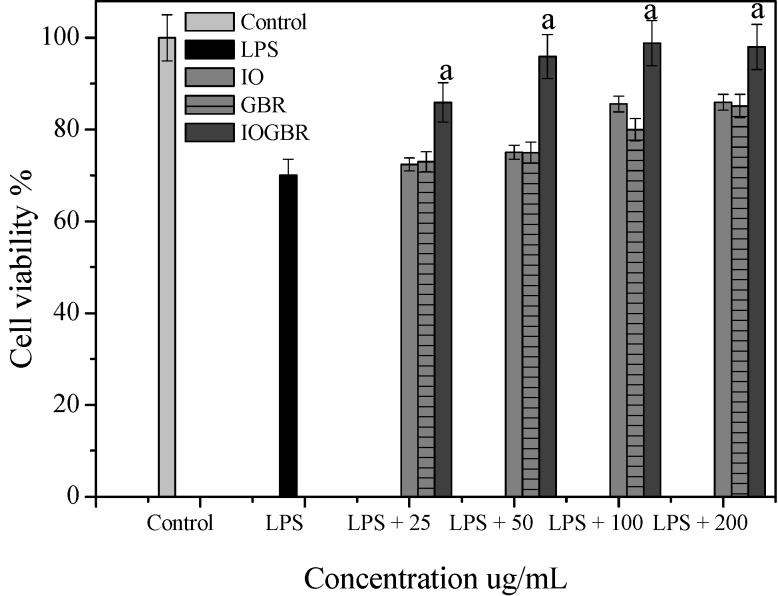
Cell viability of the extracts from *Inonotus obliquus* (IO), germinated brown rice (GBR) and *Inonotus obliquus* grown on germinated brown rice (IOGBR) on LPS induced Raw 264.7 cell.

A diet rich in mushrooms has been negatively correlated with various diseases, which are associated with inflammatory disorders. Mushrooms and their active compounds have demonstrated immunomodulatory activities by increasing the production of cytokines (IL-10, IL-12p70, and IL-12p40) by dendritic cells (DC), the activation of natural killer (NK) cells, increased production of TNF-α, IL-1, IL-6, and NO and expression of iNOS by macrophages [24]. Moreover, there is a large body of evidence that NO is involved in various types of inflammatory disorders. It acts as an anti-inflammatory agent under normal physiological conditions. On the other hand, the overproduction of NO is considered as a proinflammatory mediator under abnormal conditions that induces inflammation. As found in many chronic inflammatory disorders, a variety of stimuli-such as with LPS produced a massive amount of NO by the activated macrophages which can participate in moving towards more intense inflammatory responses. PGE_2_ is produced by mammalian tissues as a lipid mediator and has been considered as a key mediator of inflammation. Therefore, the agents, which are capable to inhibit the excessive production of inflammatory mediators such as NO and PGE_2_ in inflammatory cells, might have potential therapeutic value to inflammatory diseases. We examined the effects of IO, GBR, and IOGBR extracts on NO and PGE_2_ production by RAW 264.7 cells to evaluate whether the extracts could modulate NO and PGE_2_ production by activated macrophages. Here, NO and PGE_2_ levels in LPS-stimulated cells increased significantly compared to the control., The IOGBR markedly inhibited NO and PGE_2_ production only as shown in Figure 2a,b, supported by cell viability experiments (Figure 3), which were performed by MTT assay.

**Figure 4:**
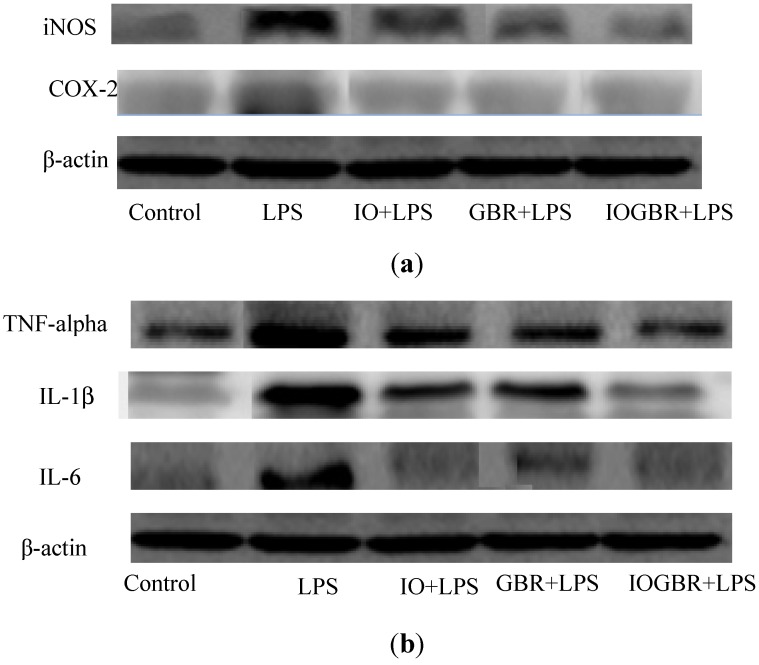
The effect of the extracts from *Inonotus obliquus* (IO), germinated brown rice (GBR) and *Inonotus obliquus* grown on germinated brown rice (IOGBR) on protein expression.

In addition, three isoforms of nitric oxide synthase (NOS) such as neuronal NOS (nNOS), endothelial NOS (eNOS), and inducible NOS (iNOS), which mainly catalyzed the production of NO. Various stimuli, such as LPS, can promote the over-expression of iNOS and produce NO mediated inflammation [25]. On the other hand, the production of prostaglandins from arachidinoic acid depends on two isoforms of COXs. COX-1 is constitutively expressed out of the two isoforms, while COX-2 is expressed by different cell types such as macrophages, endothelial cells, and fibroblast upon induction [26]. Therefore, inhibiting the overproduction of iNOS and COX-2 may be the sign of anti-inflammatory capacity of samples. In this study, we found that all the samples reduced the expression of iNOS and COX-2 in the LPS-stimulated RAW-264.7cell as shown in Figure 4a.

TNF-α, IL-1β, and IL-6 are potent pro-inflammatory cytokines usually released by macrophages and play a crucial role in initiating and sustaining the inflammatory response. IL-1β plays an important role in the initiation and enhancement of the inflammatory response to the *Helicobacter pylori* infection, and IL-6 is considered as an endogenous mediator of an LPS-induced fever [27,28]. Moreover, it is reported that several mushrooms exhibited anti-inflammatory activity by elevating anti-inflammatory IL-10 production, reducing pro-inflammatory IL-1β, IL-6, or TNF-α production, or reducing the expression of iNOS and COX-2. Here, the highest anti-inflammatory effect was detected with IOGBR, which slightly reduced TNF-α expression and markedly reduced IL-1β and IL-6 secretion in Figure 4b.

Hence, the excellent inhibitory activity of IOGBR on pro-inflammatory cytokines or iNOS and COX-2 expressions in macrophage offers a new therapeutic strategy for the treatment of inflammation. Van *et al.* [29] also found that a fraction of *Inonotus obliquus* possesses anti-inflammatory properties in a RAW 264.7 macrophage cell.

These results suggested that the extracts prevented the oxidative stress probably through their free radical scavenging, anti-lipid peroxidative and enzymatic antioxidant activities in the liver and have the potential to control diseases associated with oxidative stress or inflammation. Our findings show that the bio-activity of *Inonotus obliquus* was not reduced or changed when it was grown on germinated brown rice. The higher activity of IOGBR may be related to its higher phenolic and flavonoid contents [17]. Park *et al.* [13] reported that *Phellinus linteus* grown on brown rice contains greater amounts of some constituents including ergosterol peroxide, γ-aminobutyric acid (GABA), and β-glucan compared to *Phellinus linteus*. Our study is in agreement with that report.

## 3. Experimental

### 3.1. Materials and Chemicals

LPS was purchased from Sigma (St, Louis, MO, USA). Dulbecco’s Modified Eagle’s medium (DMEM) and 3-(4,5dimethythiazol2-yl)-2,5-diphenytetrazoleum (MTT) were obtained from Wako Chemical Co. (Tokyo, Japan). Fetal bovine serum (FBS) and antibiotics were purchased from Gibco-BRL (Gaithersburg, MD, USA). All chemicals were used without any further purification.

### 3.2. Preparation of Extracts

Germinated brown rice was purchased from a local market and IO mushroom was inoculated on germinated brown rice according to the method of Jeon *et al.* [15]. Then dried mushroom (100 g) was extracted with 70% ethanol (500 mL) at room temperature (RT) for 24 h. The extraction procedure was performed three times. Afterwards, the extracts were filtered, evaporated and then dried. The extracted sample was dissolved in distilled water (DW) and used for the experiments. 

### 3.3. Intracellular ROS Detection

Intracellular ROS formation was detected by 2¢,7¢-dichlorofluorescindiacetate (DCF-DA, Sigma), which is oxidized to fluorescent 2¢,7¢-dichlorofluorescin (DCF) by hydroperoxides. Chang liver cells were seeded 96 well black plates at the concentration of 4 × 10^5^ cells/mL for 24 h and then treated with various concentrations extracts and incubated for 1h. Then, cells were washed with serum-free DMEM for 3 times and incubated with 50 μM DCF-DA (in serum-free DMEM) in the dark for 30 min at 37 °C. After that, cells were washed with cold PBS for 3 times and resuspended in PBS. The intracellular levels of ROS were detected by measuring the mean fluorescence intensity by flow cytometry (BD FACS Calibur^TM^, Becton Dickinson, Franklin Lakes, NJ, USA). The excitation and emission were at 485 and 535 nm respectively.

### 3.4. Preparation of Liver Tissue Samples

Four-week-old male BALB/c mice (average body weighing 18.7 ± 1.6 g) were obtained from Orient Bio (Seongnam, Korea). Animals were kept in stainless steel bottom cages in a windowless room on a 12 h light/dark cycle according to the role of institutional animal care and use committee of the Konkuk University. After seven days of adaptation, we sacrificed the mice and 100 mg of liver sample was taken in six separate tubes denoted by A (control sample), B (damage sample), and C to F (treatment sample) containing 5 mL of 0.1 M Tris-HCl buffer (pH-7.4) [30,31]. The liver tissue was homogenized in all tubes, followed by the addition of 100 μg/mL of GBR, IO, and IOGBR into C, D, and E, and 10 μg/mL of ascorbic acid into the F tubes, respectively. Then, the samples were incubated at 37 °C for 30 min. H_2_O_2_ was added into all tubes except A and followed by the incubation again at 37 °C for 30 min. Then, the samples were centrifuged with 2,500 rpm at 4 °C and the supernatants were collected to perform antioxidant assays.

### 3.5. Lipid Peroxidation (LPO) Assay (Estimation Malondialdehyde Level)

MDA is one of several products formed via the decomposition of certain primary and secondary lipid peroxidation products. MDA reacts with thiobarbituric acid to form a pink chromogen (thiobarbituric acid-2-malondialdehyde adduct), which is measured spectrophotometrically. The level of LPO in different groups was measured according to the method of [32]. Briefly, tissue homogenate (200 μL), reagent blank (containing 0.8 mL of distilled water) and standard tubes containing MDA at different concentrations were mixed with 8.1% SDS (0.2 mL), acetic acid (1.5 mL) and TBA (1.5 mL). This mixture was made up to 4 mL with distilled water and kept in water bath at 90 °C for 1 h. After cooling the mixture to room temperature, distilled water (1 mL) was added and the mixture centrifuged at 3,000 rpm for 10 min. The pink color formed was measured at 532 nm against blank using spectrophotometer. The Thiobarbituric Acid Reactive Substance (TBARS) thus measured was expressed as μmoles of MDA formed/min/mg protein in tissue samples.

### 3.6. Estimation of Activity of Glutathione Peroxidase (GPx)

Triplicate samples of tissue homogenates (100 μL) were taken in separate tubes containing phosphate buffer (0.4 mL), sodium azide, EDTA and H_2_O_2_ (0.1 mL of each). Then, distilled water (1.0 mL) and GSH (0.2 mL) were added to all these tubes. The reaction was stopped by the addition of 10% TCA (0.5 mL) at 0, 1.5 and 3 min intervals in the water bath at 37 °C. Then, the tubes were centrifuged and 1.0 mL of the supernatant was collected. The blank was constituted of 1 mL distilled water. The glutathione standard was prepared in separate tubes at a concentration range of 5 to 20 μg in a final volume of 1 mL. Phosphate solution (4 mL) was added to all above tubes and then DTNB (0.5 mL) was added just before the reading and the developed color was read immediately at 412 nm against the blank using a spectrophotometer. The activity of GPx in tissues is expressed as units/mg protein. One unit of enzyme activity is the amount of the enzyme that converts 1 μmole of GSH to GSSG in the presence of H_2_O_2_/min.

### 3.7. Superoxide Dismutase Like (SOD-like) Scavenging Activity Assay

The SOD-like activities of samples were determined using the method described by [17]. Briefly, different samples (200 μL) were mixed with pyrogallol solution (200 μL, 7.2 mM in water) and 50 mM Tris-HCl buffer at pH 8.5 containing 10 mM EDTA (3 mL). The absorbance was taken at 420 nm. SOD-like activity was calculated using the following equation:

Scavenging activity (%) = 100 − [{(A − B)/C} × 100]

where A = Absorbance of sample with reagent, B = Absorbance of sample without reagent, and C = negative control (only DW + reagent).

### 3.8. Catalase Activity Assay

Cayman’s catalase assay kit was used to investigate the enzymatic activity in the peroxidation function of CAT and the assay was performed according to the manufacturer’s instructions. 

### 3.9. Determination of Cell Viability

RAW 264.7 cells (American Type Culture Collection, ATCC-TIB-71) were seeded at a density of 5 × 10^5^ cells per well in 12 well plate containing 100 μL DMED with 10% heat inactivated FBS and incubated overnight. Various concentrations of extracts (50–200 μg/mL) were dissolved in phosphate buffered saline (PBS) and applied to the cell cultures alone or with 10 μg/mL of LPS for a day. After that the cells were washed once then mixed with 50 μL of FBS free medium containing 5 mg/mL of 3-(4,5-dimethylthiazol-2-yl)-2,5-diphenyltetrazolium bromide (MTT). After 4 h of incubation at 37 °C, the medium was discarded and the formazan blue, formed in the cells, was dissolved in 100 μL of dimethyl sulfoxide (DMSO). The optical density was measured at 540 nm. 

### 3.10. Measurement of Nitric Oxide and Prostaglandin E_2_ Production

NO was measured with cell supernatant by using the Greiss reagent. The Raw cells were cultured with DMEM and 10% FBS. A total of 5 × 10^5^ cells were put into a 96 well plate and washed two times with PBS when the confluence was 80% and then cultured for at least 24 h and the samples were made into the final concentrations of 25, 50, 100, 200 μg/mL for experiments. Four hours later, LPS (final concentration 10 mg/mL) was applied into all wells except control groups well, the amounts of NO generated were measured with the supernatant 18 h later at 540 nm. The nitrite concentration was determined by extrapolation from the sodium nitrite standard curve. PGE_2_ production from endogeneous arachidonic acid was measured in cell culture supernatants with an ELISA kit according to the supplier’s instructions (EIA; Cayman Chemical).

### 3.11. Western Blot Analysis

Cellular proteins were extracted from treated and untreated cell and homogenized in RIPA lysis buffer with 1% protease inhibitor cocktail using a PRO 200 homogenizer followed by sonication with a dismembrator. Then, the homogenates were centrifuged for 15 min at 12,000 g at 4 °C. The cell debris was removed, supernatants were collected. The concentration of protein was estimated by using the Bio-Rad protein assay reagent according to the manufacturer’s instructions. A fixed amount (50 μg) of cellular protein from the treated and untreated cell extracts was separated using SDS-polyacrylamide gel electrophoresis and was electroblotted onto a nitrocellulose membrane. The immune blot was incubated overnight with a blocking solution, followed by incubation with dilution of polyclonal antibodies against iNOS, TNF-*α*, Cox-2, IL-1β, IL-6. The blots were washed twice with Tween20/Tris-buffered saline (TTBS) and incubated with diluted solutions of respective secondary antibody for 1 h at RT. The blots were washed five times with TTBS and developed under enhanced chemiluminescence.

### 3.12. Statistical Analysis

All experiments were performed in triplicate and all data were expressed as mean standard deviation (SD). Statistical analyses were performed with Graph Pad InStat. The observed differences were analyzed for statistical significance by one-way analysis of variance with Tukey’s multiple comparison as a *post-hoc* test. Differences of *p* < 0.05 were considered as significant.

## 4. Conclusions

In conclusion, the antioxidant and anti-inflammatory activities of IOGBR ethanol extract were studied and it was compared with those for IO and GBR, whereby the IOGBR showed the highest antioxidant and anti-inflammatory activities. The IOGBR extracts scavenged total intracellular ROS and MDA levels as well as increased the antioxidant enzymes activity in an H_2_O_2_-stimulated mice liver. It also suppressed proinflammatory mediators such as NO, PGE_2_, iNOS, COX-2, TNF-α, IL-1β, and IL-6 in an LPS-stimulated RAW 264.7 cell line. In our previous report, we investigated the antioxidant activity of different extracts from IOGBR by measuring the scavenging various free radicals *in vitro* as a preliminary screening study. However, the present study was aimed at investigating its anti-oxidant activities extensively along with H_2_O_2_ induced liver as well as anti-inflammatory activities with RAW cells, which were eventually compared with those for IO and GBR. Our findings suggested that the bio-activity of *Inonotus obliquus* is enhanced significantly when it was grown on germinated brown rice. This comparative study provides a novel approach to find out an excellent natural source of antioxidants and anti-inflammatory agent as a dietary supplement by growing *Inonotus obliquus* on brown rice using a well-known technique. We are deeply interested in extending this *ex (in) vivo* study to clinical trials.
